# From foreclosure to potentiality

**DOI:** 10.1177/20438206261424527

**Published:** 2026-03-02

**Authors:** Santiago del Río, Sarah Marie Hall, Elizabeth Ackerley, Laura Fenton

**Affiliations:** The University of Amsterdam, the Netherlands; The University of Amsterdam, UK; Greater Manchester Combined Authority, UK; University of Sheffield, UK

We are truly grateful and delighted for the thoughtful responses of the four scholars – Anna Barford, Valentina Cuzzocrea, Richard Ronald, and Sander van Lanen – whose reflections enrich and extend the arguments of our original paper while opening exciting new directions for empirical research and conceptual development. With our original contribution (del Río et al., 2026), we advanced the notion of ‘foreclosed futures’ to critically examine how prolonged austerity is not merely experienced as an extended phase of precarity associated with delayed transitions into adulthood – a perspective that implicitly assumes the eventual attainment of stability, albeit later in life. Rather, ‘foreclosed futures’ enables the exploration of how enduring denial of ontological security is embodied and emotionally navigated by young adults whose lived experiences have been shaped exclusively within the context of austerity.

The four commentaries engage with critical dimensions of the concept, including the potential to broaden its geographical, relational, and comparative reach, the role of the state in (re)constituting the social infrastructures that underpin security and enable conditions for hopeful futures, and its potential for reconfiguring understandings of agency and socio-political transformation. Our response, therefore, offers an opportunity to reflect further on these ideas and offer suggestions for further inspiration and ongoing dialogue.

## Geographies of foreclosed futures

The commentaries together underscore the importance of extending the conceptual reach of foreclosed futures beyond our initial focus on the European context. Barford (2026) notes that the shifts outlined in our paper are mirrored in the Global South, where converging trends among young people can be traced. Building on research in South Asia and Africa (Shankland et al., 2022), Barford argues that, without opportunities for formal work and without clear pathways towards material security, young people tend to approach loans cautiously and prefer stability-oriented financial tools. This aligns with recent empirical research that applies the concept of foreclosed futures in Spain (del Río, 2025), where persistent precarity and the erosion of ontological security give rise to a yearning for stability. Yet, this longing is not expressed through hopeful visions of the future, but rather through a ‘cruel nostalgia’ (del Río, 2025) – a melancholic attachment to a more secure past that feels irretrievably lost.

In her commentary, Cuzzocrea (2026) reflects on the potential to expand the geographical scope of foreclosed futures. She notes that this idea resonates with the concept of ‘waithood’ – a structurally imposed suspension rather than a voluntary delay in the transition to adulthood. Unlike the ‘fast-track’ transitions once typical of working-class youth in the Global North, ‘waithood’ in Global South contexts traps young people in enduring marginality. This lens is useful to illuminate how young people in the Global North increasingly experience a prolonged limbo of dead-end jobs, in-work poverty, extended dependence on family support, and an endless cycle of housing precarity. Yet, we wonder whether foreclosed futures might gesture towards something beyond waithood, while still unfolding alongside it. Perhaps it evokes an affective-temporal horizon where the act of imagining the future collapses into a condition defined by the absence of hope for stability itself.

Similarly, Ronald (2026) outlines avenues for developing a broader geographical conceptualisation of experiences of enduring precarity. He draws parallels between arguments rooted in the 2008 crisis and Japan’s 1990s financial downturn, which produced a ‘lost generation’ characterised by delayed family formation and a rise in single adults exposed to housing precarity. This insight helps extend the concept of foreclosed futures beyond young people. Our paper draws on an understanding of austerity not as a time-bound policy framework, but as a complex, lived condition – socially embedded and embodied – that can persist indefinitely and be experienced as never-ending (Hall, 2022). This perspective can help illuminate how precarity continued to shape the life trajectories of adults entering mid-life, including ageing millennials whose paths were affected by the 2008 crisis. It also encourages research into how austerity disrupts geographically contingent ideals of life transitions and notions of the ‘good life’, carrying broader socio-political implications that may be reshaping personal and collective imaginaries of progress.

Such an approach can deepen our understanding of the geographical, political, and emotional implications of pernicious socio-economic dynamics, foreclosing hope and potentiality. This connects with a recurring theme in the commentaries: the role of the state in both enabling and constraining the sense of potentiality that underpins future imagining and future-making.

## Reimagining welfare

In his response, Van Lanen (2026) introduces the concept of ‘foreclosed spaces’ – understood as the erosion of public infrastructures and services – and explores how it reshapes the geographies of informal care. He notes that foreclosed spaces arise when young people are priced out of neighbourhoods with accessible care services and pushed away from family caring networks. As public and familial care decline, private providers fill gaps for those who can pay, while low-income households face limited options. As noted in our original paper, rising housing costs also trap young people in – or push them back to – the parental home, fostering feelings of stagnation and inadequacy.

With contemporary policy regimes oriented towards asset inflation and social and direct wage repression (Adkins et al., 2020), families not only absorb welfare-state retrenchment (Hall, 2016) but also invest in housing as a key asset to secure ontological security, reproducing family-based class inequalities. As Ronald (2026) notes, when parents are renters or own low-value homes, their ability to support children is limited, leaving their children exposed to enduring experiences of precarity. Furthermore, as we discuss in the original paper, the deterioration of the welfare state, which gives rise to ‘familialisation’, excludes and leaves unprotected those unable to rely on family support, reproducing intergenerational inequalities.

Interrogating the political ramifications of the austere state, Barford (2026) poses a key question: ‘As austerity undercuts the functioning of the state, will subsequent generations never learn what a state could provide?’ We recognise that enduring austerity can reinforce anti-statism and fuel the rise of authoritarian populisms – particularly among young men – who are increasingly drawn to far-right political movements (Cokelaere, 2024). Austerity can equally lead to disengagement due to diminished resources for participation (Harrison, 2020).

However, conditions of persistent ontological insecurity can also stir nostalgia for an idealised welfare state. In the first paper, operationalising the idea of foreclosed futures, del Río (2025) found that in a context of extreme youth precarity in Spain, young people did not imagine security by projecting themselves into better futures but through the experiences of upward mobility of their parents and grandparents. In this context, far from withering away, the welfare state becomes a powerful, yet impossible object of desire. As del Río notes (del Río, 2025: 16),

On the one hand, post-war notions of the good life continue to function as the grammar through which security is imagined and understood. On the other, this grammar is articulated solely in the past tense, no longer as a cruel attachment fuelling hope for better futures, but as a cruel nostalgia for a past irretrievably lost.

Perhaps this sense of cruel nostalgia helps explain the contradictory stance of far-right populist movements, which oscillate between libertarian, anti-statist rhetoric and a romanticised, exclusionary vision of welfare chauvinism that underpins a politics of ‘making nations great again’. Welfare chauvinism gestures towards a deep-seated yearning for ontological security – conditions that could equally be mobilised to cultivate inclusive political possibilities. Rather than simply invoking past experiences of the post-war welfare state – experiences marked by gender, racial, and sexual exclusions and imperial legacies – a collective and educated sense of hope can be ignited through the imaginative construction of post-austerity welfare models. This exercise of imaginative state deconstruction and reconstruction is not only vital for restoring the universal foundations of the vanishing welfare state. It is also crucial for envisioning a welfare model that provides ontological security not through a singular way of living, or an exclusionary, standardised life course, but across plural life course trajectories.

## From foreclosure to potentiality

It is at this juncture that the analytical salience of foreclosed futures can become especially powerful. We suggest that foreclosed futures can serve as a conceptual lens for understanding the affective and temporal conditions generated by austerity, while also opening space for imagining alternative futures. In this vein, Apostolopoulou and Liodaki (2025) show that enduring precarity can also be catalyst for grassroots mobilisation aimed at safeguarding and (re)producing public spaces, green areas, and infrastructure while also fostering cross-community solidarity. In various ways, the four commentaries underscore the importance of examining how personal and social transformations can emerge from materially austere conditions. We conceptualise enduring austerity not merely as a policy regime exacerbating hardship, inequality, and material insecurity but as an embodied and affective process that constrains the capacity to imagine futures otherwise.

This perspective offers a conceptual and analytical entry point into the intimate aspirations, longings, and dreams shaped by austerity, along with the affective-temporal formations through which they are articulated. In this sense, the concept of foreclosed futures serves not as a theoretical terminus but as a generative point of departure for research that seeks to uncover the austerity's dashed dreams and to illuminate the longings of those experiencing precarity as an enduring condition. As Cuzzocrea (2026: 3) underscores, ‘the experience of having a foreclosed future conveys the idea of something that is rightfully theirs but remains out of their possession’.

Building on this insight, we argue that research and teaching informed by this concept holds the potential to experiment with methodologies and forms of co-creation that not only analyse how futures are denied but also enable both young and not-so-young people to articulate and envision the futures they desire. Ultimately, this approach can generate empirically grounded knowledge and narratives that foster alternative future imaginaries, challenge foreclosure and open new horizons of hope. In a context where austerity dispossesses increasing numbers of people of their capacity to imagine other futures – and with it, their very sense of potentiality and possibility – creating spaces for future imagining becomes a profoundly political act.
